# The Impact of Vaccination and Antiviral Therapy on Hepatitis B and Hepatitis D Epidemiology

**DOI:** 10.1371/journal.pone.0110143

**Published:** 2014-10-14

**Authors:** Ashish Goyal, John M. Murray

**Affiliations:** School of Mathematics and Statistics, University of New South Wales, Sydney, Australia; Centers for Disease Control and Prevention, United States of America

## Abstract

The major cause of liver cancer around the globe is hepatitis B virus (HBV), which also contributes to a large number of deaths due to liver failure alone. Hepatitis delta virus (HDV) is as potentially alarming as HBV since life threatening cases are 10 times more likely with HBV-HDV dual infection compared to HBV monoinfection. So far, there is no established effective treatment against HDV and the only preventive action suggested by the World Health Organization is to introduce HBV vaccination for children immediately after birth (newborns) and thus reduce the available pool for HDV infection. Here the main objective is to understand the complex dynamics of HBV-HDV infection in a human population that can inform public health policy makers on the level of different preventive measures required to eliminate HBV and HDV infections. Model simulations suggest that HBV vertical transmission and HBV vaccination rates for newborns are instrumental in determining HBV and HDV prevalence. A decrease in HBV prevalence is observed as vaccination coverage increases and it is possible to eradicate both HBV and HDV using high vaccination coverage of ≥80% in the long term. We further found that HDV presence results in lower HBV prevalence. An application of our model to China revealed that vaccinating every newborn in China will further prevent 1.69 million new infections by 2028 as compared to the current 90% vaccination coverage. Although, higher vaccination coverage of newborns should eliminate both HBV and HDV over a long time period, any short term strategy to eradicate HDV must include additional preventive measures such as HBV adult vaccination. Implementation of HBV adult vaccination programs at a rate of 10% per year over 15 years will further prevent 39 thousand new HDV infections in China by 2028 as compared to HBV vaccination programs solely for newborns.

## Introduction

Almost two billion people have been infected with hepatitis B virus (HBV) at some stage of their lives, with 400 million chronically infected. Chronic infection can lead to cirrhosis and liver cancer resulting in 0.5–1.2 million deaths a year [1]–[5]. Approximately 5% (18 million) of HBV infected individuals are also infected with hepatitis delta virus (HDV) [6]. Individuals infected with HDV have a high mortality rate of 2–20% in 5–10 years which is ten times higher than for those with HBV monoinfection [7]–[9]. HBV is prevalent in Asia, sub-Saharan Africa, the Amazon Basin, parts of the Middle East and some countries in Eastern Europe [10]–[13], while HDV is endemic in the Amazon Basin, the Mediterranean Basin, some parts of Asia and Central Africa [10]–[13].

The modes of transmission for HBV are sexual contact and other forms of blood exchange (parenteral exposure-horizontal transmission) as well as infection transmitted from mother to child during birth (perinatal-vertical transmission). Young children infected with HBV after perinatal transmission are asymptomatic and 95% become chronic carriers of which 25% die in adulthood from cirrhosis or liver cancer. Only 3–5% of adults progress to chronic HBV infection [14], [15]. HDV uses the same transmission modes (except for perinatal) but its infectivity is constrained by relying on HBV for its replication, infectivity and transmissibility. HDV can replicate inside a host cell that is not infected with HBV but cannot assemble and release its virions to infect other cells or other people. The rate of conversion from acute to the chronic stage of HDV infection is also very high, 5 times more than in those with HBV monoinfection [16], [17] and the incidence of liver failure is much higher than for individuals with HBV monoinfection. HBV transmission is also less likely from someone who is infected with HDV [7], [8], [18].

Mathematical models have been used previously to determine the spread of HBV infection in a population and some also modelled vaccination programs [19]–[23]. In the case of HDV however, only one model has been proposed so far by Xiridou et al. [16] for the transmission dynamics of HDV in a general population infected with HBV. While this model incorporated horizontal transmission of HBV and HDV infection, it did not include vertical transmission of HBV which is a major source of new infections despite the availability of a vaccine [24]. In this study, we specifically include vertical HBV transmission and the effect of HBV vaccination for children immediately after birth on HBV and HDV prevalence. The study of the impact of HBV vertical transmission on HDV prevalence and how HBV vaccination affects HDV prevalence will be among the major goals of this article. The model was specifically applied to the HBV and HDV epidemics in China as approximately 7% of the population was infected with HBV in 2006 [25]. We found that higher vaccination coverage will eradicate both HBV and HDV in the long term, but that it was vital to eliminate HBV infection in children through HBV vaccination. The level of coverage required to eliminate either one of the viruses or both viruses was also determined which can be highly useful to public health policy makers. The presence of HDV also provided assistance in the reduction of endemic HBV. We found that as the prevalence of HDV increases, HBV prevalence decreases. However, this reduction in HBV is followed by an undesirable increase in liver cancer cases and subsequent deaths.

## Materials and Methods

### Mathematical Model

A mathematical model was constructed based on the characteristics of HBV/HDV transmission dynamics adopted by Xiridou et al. [16]. In addition to that model, we also incorporate the following features:

1. HBV and HDV horizontal transmission along with HBV vertical transmission are included in the model.
2. A recovered group is included.
3. We included birth and death rates relevant to an expanding population.
4. Vaccination against HBV immediately after birth is included.
5. Disease-induced mortality rates in the chronic phase of HBV and HDV infection have also been incorporated.

**Figure 1:**
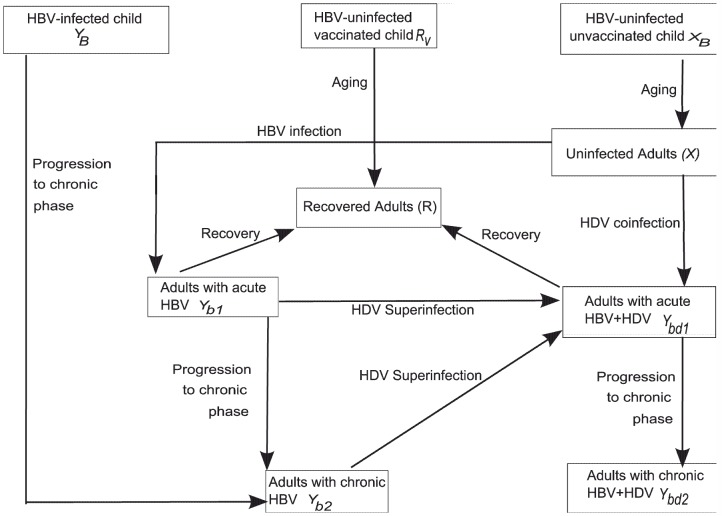
Schematic representation of HBV/HDV transmission dynamics in a population.

The total population is divided into nine compartments where the variables 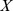 represent susceptible individuals, 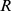 resistant to both HBV and HDV and 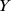 infected with one or both viruses: uninfected adults (aged 15 years and older) (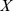), uninfected and unvaccinated children (aged 0–14 years) (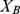), children vaccinated against HBV immediately after birth (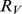), individuals born with HBV infection (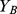), HBV infected adults in the acute phase (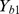) and chronic phase (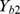), HBV-HDV dually infected adults in the acute phase (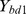) and chronic phase (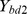) and fully recovered adults from either the acute phase of HBV or HBV-HDV infection (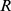). Uninfected adults become infected with HBV depending upon the sexual contact with HBV mono-infected adults or dually infected adults. Infection then passes through two phases namely, an acute phase and a chronic phase. The clinical course of HDV infection follows two paths: (i) HDV superinfection (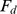), in which a previously HBV infected individual is infected with HDV, and (ii) HDV coinfection (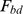), in which an individual is infected with HBV and HDV at the same time. During HDV coinfection, there is a low risk of development of chronic infection (5–10%) while during HDV superinfection almost 70–80% individuals will progress to chronic infection [26]–[29]. HDV also passes through two phases as well. A detailed description of the transmission dynamics between different compartments is shown in Figure 1.

We assume that a proportion (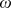) of children are successfully vaccinated immediately after birth and these vaccinated children move to the immunized class 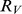. Children born with HBV (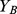) are asymptomatic and do not go through the acute phase of HBV infection [14], [15] and it takes 20–30 years before they progress to the chronic stage of HBV infection [30]. For simplicity however, we assume that individuals born with HBV progress to adulthood and the chronic phase after an approximate 14 year duration.

We included two classes of susceptibles, children (aged 0–14 years) and adults (aged 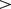 14 years), as children have higher mortality rates at very young ages. There is a very low risk of passing HDV from mother to child during birth [31] which we do not include in the model. We also assume that once either infection (HBV or HDV) progresses to the chronic phase, there is a very low chance of spontaneous recovery from the infection. The full system of the model equations is given by, 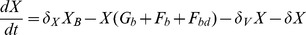


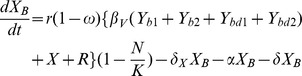


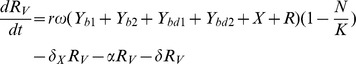


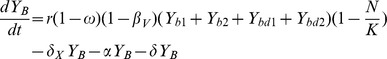


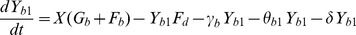


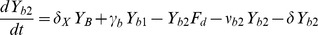


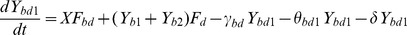


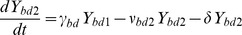


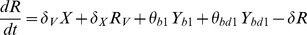
(1)


The total population is denoted by 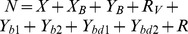 and is assumed to grow logistically in the absence of any infection.

We define the per capita risks of each infection similar to those of Xiridou et al. [16]

1. Infected only with HBV from an individual with HBV monoinfection: 
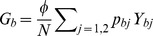
,
2. Infected only with HBV from a dually infected individual: 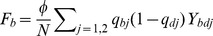,
3. Superinfected with HDV: 
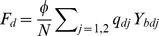
,
4. Coinfected with HBV and HDV: 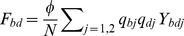,

The parameters 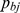, 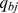, and 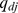 (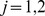) correspond to probabilities of HBV and HDV horizontal transmission while 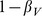 is the probability of vertical HBV transmission with 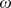 being the effectiveness of vaccination. The parameter 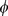 describes the rate of partner change. Demographic parameter values such as birth rate 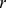, maximum carrying capacity 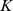 and death rate 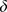 were determined for China. Parameters such as 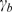, 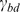 and 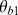, 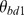 represent the progression rate of HBV and HDV infection in the chronic phase, and the recovery rate of acute HBV and HDV infection respectively. The parameters are described along with their values in Table 1.

**Table 1:** Parameter values and their definitions.

Parameter	Interpretation	Value	Reference
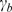	Rate of progression from acute to chronic HBV infection	0.4/person/year	[16], [19], [23]
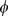	Rate of partner change	1.64 partners/year ( 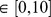 )	[16]
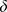	Death rate in population	0.007/year	[42]
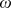	Vaccination coverage in children immediately after birth	0.9 ( 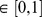 )	[21], [42] a
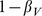	Probability of passing HBV infection from mother to child during birth	0.9 ( 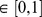 )	[22]
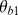	Recovery rate of adults from acute HBV infection	3.6/person/year	[16], [19], [23]
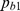	Probability of HBV transmission from an individual in the HBV acute phase	0.46	[16], [23]
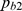	Probability of HBV transmission from an individual in the HBV chronic phase	0.65 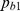	[16], [23]
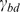	Rate of progression from acute to chronic in dually infected	2/person/year	[16], [23]
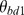	Recovery rate of adults from acute dual infection	2/person/year	[16], [23]
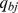	Probability of HBV transmission from dually infected individual at stage 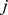 = 1,2	0.71	[16], [43]
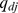	Probability of HDV transmission from dually infected individual at stage 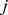 = 1,2	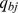	[16]
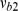	Disease induced mortality rate in chronically HBV infected individuals	0.0013/year	[7]–[9]
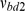	Disease induced mortality rate in chronic dual infection	0.013/year	[7]–[9]
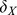	Rate of maturation to adult aged 14+ years for uninfected children and children born with HBV	1/14/year ( 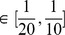 )	[44]
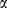	Extra-mortality rate during childhood (for China)	0.01/year	[42]
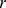	Rate of growth of population in China	0.019/year	[42]
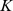	Maximum Carrying Capacity of the population in China	3.8e+09	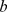
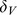	Adult vaccination coverage	0/year	

*^a^*vaccination started in China in 1992 with a coverage of 20%. In 2003, vaccination coverage reached 90% and continued to increase. We assumed it to be a constant coverage of 90% from the beginning of the vaccination programs.

*^b^*obtained after fitting the total population (using non-linear least squares and assuming logistic growth) to population data in China from [45], for the period 2003–2011. This procedure reproduced the data of the total population well.

### Procedure and Methodology

We tested the applicability of the model (1) by reproducing HBV and HDV prevalence in China since the introduction of vaccination programs in 1992. For this purpose, we first estimated the value of the rate of partner change (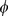). The sexual contact rate can vary for China from the value based on previous estimates (given in Table 1) and therefore, needs to be estimated for the China-specific simulations. Other parameters were kept constant during this estimation (from Table 1) because horizontal transmission probabilities per contact with an infected individual of both viruses as well as recovery rates should be universal parameters. The estimation was carried out by conducting a non-linear least squares fitting to the data derived from [25], [32]–[34]. Specifically we commenced the model simulations at 1992 literature estimates of prevalence levels of: HBV in the total population, HBV in children, and HDV among HBV infected individuals. We used the nonlinear optimisation routine ‘lsqnonlin’ in Matlab R2012a to fit the model simulations to 2006 literature estimates of HBV in the total population and among children, and also to estimates of HDV among HBV infected individuals in 2011. Using this nonlinear optimisation routine, we also estimated the sexual contact rate for China.

Estimates of steady state prevalence were obtained by running the model from 1992 for 3000 years with parameter values derived from Table 1. Sensitivity analyses were also performed with Latin Hypercube Sampling assuming the transmission probabilities (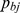, 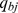, 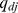; 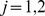) were fixed as given in Table 1 but with the following ranges for the parameters: the rate of partner change 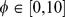, the progression rate of children into adulthood 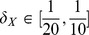, the HBV vaccination coverage in children immediately after birth 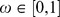 and the vertical transmission probability 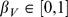. All calculations were performed in Matlab R2012a, The Mathworks Inc., Natick MA.

## Results

The SIR model (1) of HBV and HDV incorporates horizontal transmission of both viruses, as well as vertical transmission of HBV. It has four steady states corresponding to: 1) zero population, 2) a population uninfected by either virus, 3) a population only infected with HBV, and 4) a population where both viruses are endemic.

### HDV infectivity affecting HBV prevalence

**Figure 2:**
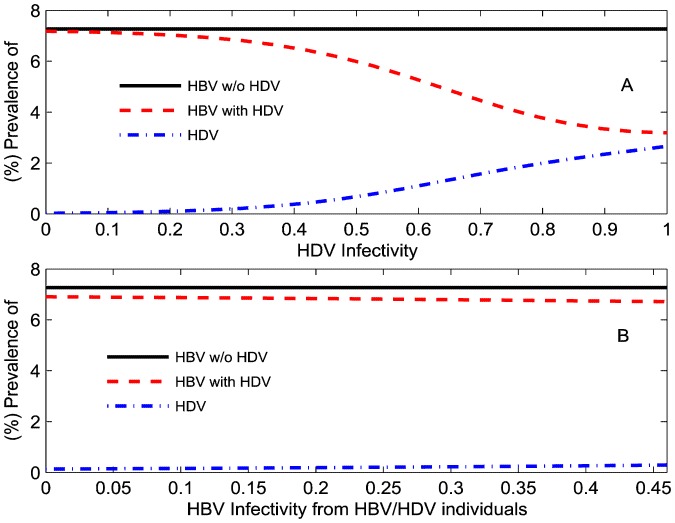
How one virus infectivity impacts the other. (%) HBV and (%) HDV prevalence in the total population during HBV mono-infection (solid black line) and dual HBV-HDV epidemics (dotted red line and dashed dotted blue line) at 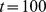 years relative to: (A) values of infectivity of acute HDV infection (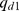) on the x-axis with 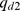 also varying as 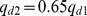; (B) levels of suppression by HDV on the transmission of HBV in those dually infected individuals (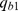) on the x-axis with 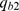 also varying as 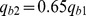.

Since there is uncertainty around the individual transmission probabilities, we investigated the effect of different HDV transmission probabilities (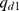 and the linked parameter 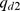) on HBV and HDV prevalence in the total population after a time of 100 years (which is before the dynamics have reached equilibrium but which provided wider variation for prevalence across the parameter values investigated in these sections) assuming 90% HBV vaccination coverage at birth and other parameters as given in Table 1 (Figure 2(A)-(B)). As HDV infectivity 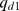 increases HBV prevalence decreases to the point where it almost equals HDV prevalence. At this point HDV is sufficiently high to ensure virtually every individual who is HBV infected is also HDV infected. Lower HBV prevalence is then a consequence of the lower probability of HBV infection from a dually-infected individual than from a mono-infected individual, which results from suppression of HBV viremia with dual infection [35]. This impact of HDV on HBV prevalence is consistent with previous HDV modeling [16].

### Suppression of HBV infectivity due to HDV in dually infected individuals

It is known that HDV suppresses HBV infectivity in dually infected individuals but the degree of suppression is uncertain. Therefore, we simulated our model for different levels of suppression of HBV infectivity in dually infected individuals (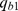 and 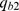) ranging from 0 to 100 percent. Higher HDV infectivity results in lower HBV prevalence, as observed above. Although a higher HBV infectivity will increase HDV prevalence, its effect is relatively small (Figure 2(C)-(D)).

We also notice that irrespective of the degree of suppression of HBV infectivity by HDV in dually infected individuals, HDV will result in a lower HBV prevalence as compared to HBV mono-infection epidemics. The increase in HBV or HDV infectivity in dually infected individuals results in a higher number of acute dual infections. A person with acute dual infection has a five times higher probability of entering the chronic phase compared to a person with acute HBV monoinfection. Furthermore the probability of HBV transmission is suppressed in dual infection populations compared to HBV monoinfection. This gives rise to a much lower HBV prevalence in dual infection populations.

We extended the model simulations, as shown in Figure 2 for HBV and HDV equilibrium prevalence at 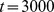 years, and estimated the critical value of HDV infectivity below which we achieve HDV eradication. With 90% HBV vaccination coverage, the critical value of HDV infectivity is given by 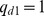 with 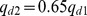. This value is very sensitive to different rates of HBV vaccination coverage. For no vaccination coverage (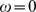), the critical value of HDV infectivity is given by 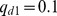 with 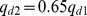. With 90% vaccination coverage, HBV is eradicated for all values of HDV infectivity whereas HBV persists irrespective of the level of HDV infectivity without HBV vaccination. These observations will allow policymakers to determine the reduction required in HDV infectivity through future therapies to eradicate HDV, under no or very low HBV vaccination coverage.

### Vaccination coverage and perinatal transmission probability affecting HBV and HDV dynamics

**Figure 3:**
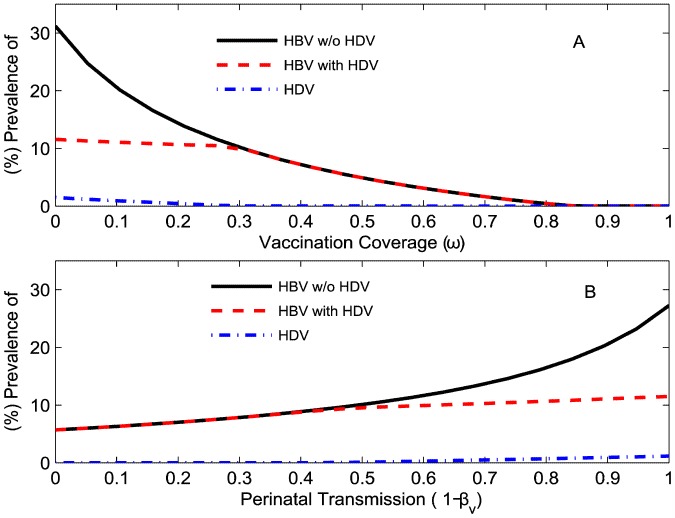
How HBV vertical transmission affects HBV and HDV prevalence in a population. Equilibrium (%) HBV and (%) HDV prevalence in the total population during HBV mono-infection (solid black line) and dual HBV-HDV epidemics (dotted red line and dashed dotted blue line) relative to: (A) vaccination coverage at HBV perinatal transmission probability 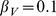; (B) perinatal transmission rate at 10% vaccination coverage (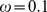).

Depending upon the level of HBV vaccination coverage, there can be different outcomes for HBV and HDV equilibrium prevalence. HBV and HDV are both eradicated if vaccination coverage is very high while only HBV will exist if vaccination coverage is moderate (Figure 3(A)-(B)). At low vaccination coverage, both viruses will be present in the population. HBV prevalence in a HBV-monoinfected population was higher than or equal to levels in a population when both viruses existed, as a result of HBV infectivity suppression by HDV in dually infected individuals [35].

The impact of different HBV perinatal transmission probabilities can be seen in Figure 3(C)-(D). Even if the HBV perinatal transmission probability becomes zero (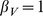), steady state HBV prevalence will still be positive. This was observed simply because zero vertical transmission probability does not induce immunity in children against future infections through horizontal transmission during adulthood. On the other hand, high vaccination coverage can achieve zero steady state HBV and HDV prevalence because it provides immunity against any possible future infections including horizontal transmission.

In summary, HDV prevalence increases as the vaccination coverage decreases and both HBV and HDV prevalence increase rapidly with the probability of perinatal transmission.

### Antiviral therapy impact on HBV and HDV prevalence

There are several antiviral therapies that reduce the replication of HBV and HDV in a host and therefore reduce the infectivity of both viruses in the population. Interferon (IFN) therapy inhibits HBV replication and is an established treatment for HBV but the results of IFN for HDV treatment have varied widely with its duration and dosage [36]. Additionally HDV antiviral therapies, such as with prenylation inhibitors, are currently under investigation through in vitro and in vivo studies in animals [37]–[39]. Therefore, in this section we simulate the impact of these therapies on HBV and HDV prevalence.

**Figure 4:**
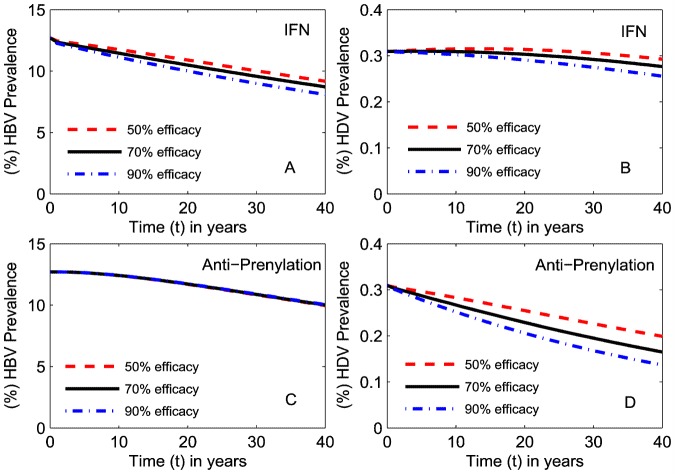
Impact of different antiviral therapies on HBV and HDV prevalence. (%) HBV and (%) HDV prevalence in the total population relative to time in years for: (A)-(B) IFN therapy reducing HBV infectivity alone in HBV mono-infected, HBV-HDV dually infected individuals and vertical transmission probability when introduced at time 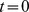 in the simulation: (C)-(D) prenylation inhibitor therapy reducing HDV infectivity alone when introduced at time 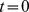 (% efficacy of a treatment is equivalent to the % reduction in infectivity).

Ignoring the risk of development of drug resistant virus, there is little difference in the impact of HBV therapy of a range of efficacies on both HBV and HDV prevalence (Figure 4(A)-(B)), each producing a slow decline in HBV prevalence and a less marked decrease in HDV prevalence. As would be expected, specific prenylation inhibitor (anti-prenylation) therapies will have a greater impact on HDV but no impact on HBV prevalence. As prenylation inhibitor simulations were carried out assuming 90% HBV vaccination coverage in children immediately after birth, the observed decrease in HBV prevalence was the result of HBV vaccination programs and not anti-prenylation therapies (Figure 4(C)-(D)). A decrease in HDV prevalence was achieved by both IFN and prenylation inhibitor therapies with greater effect shown by anti-prenylation therapy. A decrease in HBV prevalence was only observed with IFN therapy. This suggests that the eradication of HDV is coupled with the eradication of HBV but not vice-versa.

### Sensitivity Analysis

We also conducted sensitivity analyses for the HBV and HDV transmission model in order to determine the most influential parameters relative to prevalence of HBV and HDV. Latin Hypercube sampling was employed to derive 1000 test samples assuming uniform distributions of the parameters for the rate of partner change 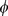, the progression rate of children into adulthood and sexual activity 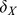, the HBV vaccination coverage in children immediately after birth 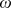, and the vertical transmission probability 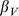 with ranges specified in Methods. We selected only these 4 parameters as the remaining parameters should be equivalent across all countries and have been estimated in the literature (Table 1). Parameters in the order of their influence on the HBV and HDV equilibrium prevalence were then found using partial rank correlation (PRCC, results are summarized in Table 2).

**Table 2:** Sensitivity analysis of the parameters representing the rate of children maturing into adulthood and sexual maturity 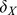, the HBV vaccination coverage in children immediately after birth 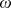, the vertical transmission probability 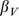 and the rate of partner change 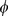 on HBV and HDV equilibrium prevalence.

Partial rank correlation coefficients(PRCC)				
PRCC	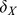	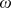	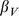	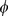
HBV	−0.0041	−0.9207	−0.3283	−0.0360
HDV	0.0271	−0.8865	−0.1476	0.8584

We found that the HBV vaccination program as determined through the parameter 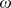 is the most influential on the prevalence of both diseases. The second most influential parameter for HBV was found to be the vertical transmission probability 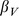 followed by horizontal transmission (the rate of partner change, 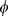). For HDV equilibrium prevalence, the second most influential parameter is horizontal transmission followed by the vertical transmission probability of HBV. The above analysis suggests that the role of vertical transmission of HBV is more important than HBV and HDV horizontal transmission in deciding the fate of HBV and HDV prevalence. Therefore, interventions affecting HBV vertical transmission such as HBV vaccination in children immediately after birth will help the most in eradication of HBV and HDV.

### Reproduction of the dynamics of HBV and HDV prevalence after the start of vaccination programs in China

In this section, model (1) was employed to reproduce the reduction in HBV and HDV prevalence in China since the introduction of HBV vaccination programs in 1992. It should also be noted that HBV prevalence has previously been modeled in China but did not include HDV and only simulated acute HBV prevalence [21], [22]. Since the total HBV prevalence was much higher than accounting for acute HBV alone, a better prediction is required [25], [32]–[34], [40]. From the available literature, we were able to derive few data points. These contained initial prevalence for each of the epidemics in 1992 that also served as initial values in the model simulation. The hepatitis B surface antigen (HBsAg) prevalence in the total population in a national survey was estimated to be 9.75% in 1992 and 7.18% in 2006 [25]. Similarly, HDV prevalence in HBV infected individuals was found to be 0.8–12% (mean 6.4%) in different provinces of China in the 1990's [40]. Shen et al. suggested a lower prevalence of HDV in HBV infected individuals in China (lower in comparison to <6% prevalence in other parts of the world) [40]. Since HDV prevalence in HBV infected individuals in China in 1992 is unknown, we used this observation and assumed HDV prevalence to be 3.2% at the commencement of HBV vaccination in 1992 (range of 0% to 6.4% HDV prevalence [40]). HDV prevalence in HBV infected individuals in 2011 and HBV prevalence in children in 1992 was found to be 1.2% and 10% respectively [32]–[34]. Liang et al. estimated HBV prevalence in young children (less than 5 years) to be 1.51% and 4% in 5–14 years through samples collected in 2006 [25]. The sampling proportion was 1∶7 between age groups <5 years and 5–14 years in the survey. In view of the large difference in the sample proportions, estimated HBV prevalence in the 0–14 year age group can be approximated to 4%, equivalent to the 5–14 years age group prevalence. Under no vaccination coverage during 1979–1992, HBsAg prevalence in the total population increased 1% from 8.75% to 9.75% [41]. We therefore assumed that HBsAg prevalence would have increased 1% from 9.75% in 1992 to 10.75% in 2005 if vaccination programs were not introduced in 1992 in China.

Vaccination coverage has varied across different provinces of China since the introduction in 1992 and we assumed 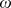 to be a constant 90% in the model simulations. Similarly, the vertical transmission probability 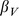 was also kept constant [22]. The sexual contact rate can vary across different countries from the value given in Table 1 and therefore, we choose 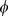 as one of the variables in fitting model simulations to the data. The few available data points do not allow us to fit too many parameters and therefore, we choose 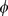 as the only variable in the fitting procedure, since the analysis above determined the model outcome was also highly sensitive to this parameter. We minimize the sum of squares error calculated by the difference in HBV prevalence in the total population under 90% vaccination coverage (in 2006), HDV prevalence in HBV infected individuals (in 2011) and HBV prevalence in children (in 2006) against their respective data points as specified in the last paragraph. HBV prevalence in the total population also increased by 1% in absolute terms between 1979 and 1992 when no vaccination was provided. Therefore we additionally required model simulations without any vaccination to increase by this same amount over 13 years from the commencement of simulations. From this procedure, we estimated 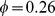 with the other parameters given in Table 1.

**Figure 5:**
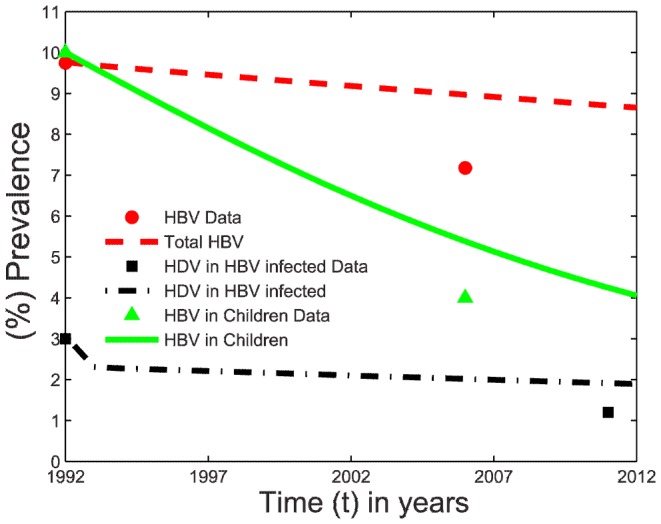
Application of the model to China. (%) HBV prevalence in the total population, (%) HBV prevalence in children and (%) HDV prevalence in HBV-infected individuals after the introduction of vaccination programs in 1992 in China. Model simulations (lines) and data (markers) obtained from [25], [32]–[34], [40].

We see from Figure 5 that our model simulations generally agree with the observed pattern of decrease in the prevalence of HBV and HDV in China, although simulated values decay more slowly than estimated from the data. The generally higher estimate of HDV prevalence among HBV infected individuals than the model initially estimated led to an initial decrease in simulations of this value. We further simulated our model assuming no HBV vaccination programs were introduced in 1992 and found that HBV prevalence increases but not up to the expected rise of 1%.

### Impact of additional preventive measures on HBV and HDV dynamics in China

**Figure 6:**
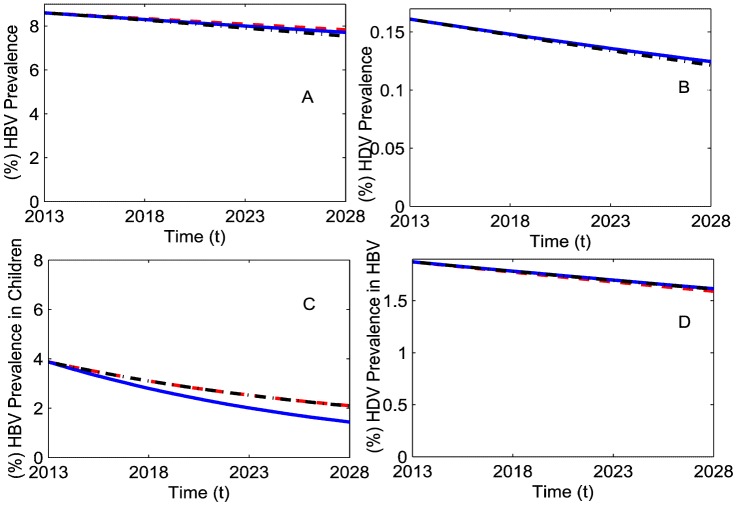
HBV and HDV prevalence under additional preventive measures in China in the next 15 years. (A) (%) HBV prevalence in the total population, (B) (%) HDV prevalence in the total population, (C) (%) HBV prevalence in children (0–14 years old), and (D) (%) HDV prevalence in HBV infected individuals. Markers were set as for 90% vaccination in children immediately after birth (red dashed line), 100% vaccination in children immediately after birth (blue solid line) and 10% adult vaccination along with 90% vaccination in children immediately after birth (black dashed dotted line).

It is clear that HBV vaccination programs have helped the reduction of HBV and HDV prevalence. But it is of interest to determine what enhanced vaccination programs including new adult vaccination programs (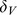) will affect HBV and HDV prevalence in the next 15 years (by 2028) and which will be better strategies in the short term prevention of HBV and HDV incidence. Therefore, we simulated our model under three scenarios, (i) 90% HBV vaccination coverage in children immediately after birth and no HBV adult vaccination (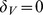), (ii) 100% HBV vaccination coverage in children immediately after birth and no HBV adult vaccination (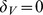) and, (iii) 10% adult HBV vaccination per year (i.e., 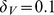) combined with 90% vaccination coverage in children immediately after birth. We calculated the impact of each preventive measure starting in 2013 on HBV and HDV prevalence by 2028 (Figure 6).

Continuing with 90% vaccination coverage in children immediately after birth (red dashed line) will further reduce HBV prevalence in the total population by 0.78% in absolute terms (10.01 million) by 2028. Increasing this coverage to 100% (blue solid line) will further decay HBV prevalence by 0.13% (Figure 6 (A)). Given the large population of China (1300 million), a 10% increase in vaccination coverage in children immediately after birth could prevent 1.69 million new HBV infections in the next 15 years. The same increase in vaccination coverage in children will also decrease HBV prevalence in children (0–14 years old) by approximately 0.65% by 2028 (Figure 6(C)). The adult HBV vaccination scenario (black dashed dotted line) further reduced HBV prevalence by 0.16% as compared with 100% vaccination coverage in children. Figure 6(D) depicts that HDV prevalence in HBV infected individuals will increase upon the introduction of any new intervention in addition to the current 90% HBV vaccination coverage in children. From Figure 6(B), we note that introduction of adult HBV vaccination will marginally reduce HDV prevalence in comparison to the other interventions which means there will be 39 thousand fewer HDV infections (Figure 6(B)).

## Discussion and Conclusion

The model produces results consistent with the decreasing pattern of HBV and HDV prevalence in China, but not exactly so for several reasons. The lower level of HBV prevalence achieved in China as compared to our model simulations may be a result of improved levels of education, awareness about unprotected sex or injecting unsafe practices, better health care facilities, and the single child policy [25] with these behavioral changes not included in the model. Differences in data and simulated values of HBV prevalence in children can also be partly explained by the exclusion of the 3% high risk population in the data [25].

We found that increasing vaccination coverage will have a huge impact on HBV prevalence alone but the introduction of adult vaccination will have the greatest impact on both HBV and HDV prevalence. This indicates that short term HDV eradication plans in China will require more efforts than just an increase in HBV vaccination coverage in children.

The model analysis indicated that in the presence of vertical transmission, both HBV and HDV can be eliminated depending upon the level of vaccination coverage, but over a longer timeframe than the 15 years simulated in Figure 6. In the case when HBV vaccination programs were not able to eliminate HDV, they were still helpful in lowering HDV prevalence. This result is somewhat similar to the observed decline in HDV prevalence in Italy or Taiwan after the introduction of HBV childhood vaccination programs [6]. This is also important because with high coverage vaccination programs, we can virtually eliminate HBV vertical transmission and consequently, it becomes possible to eradicate HBV and HDV in the long term. This shows the importance of HBV vertical transmission on the spread of both HBV and HDV in a population. While high vaccination coverage was required to eradicate HBV from the population, less effective vaccination was capable of eliminating HDV. In countries with high HDV prevalence, the model has the potential to determine the minimum rate of vaccination coverage that will help these countries to achieve HDV control even with limited resources.

When HDV infectivity is very low we determined that HBV remains endemic at almost the same level as with HBV monoinfection. At higher HDV infectivity, both HBV and HDV persist but here HBV prevalence is lower than for HBV monoinfection. This result contrasts with the findings of Xiridou et al. [16], which concluded that if HDV infectivity is sufficiently low, then HBV prevalence remains at a lower level in dual HBV-HDV epidemics than in HBV monoinfection; otherwise, HDV results in a higher HBV prevalence than HBV monoinfection. We found that this might be true for horizontal transmission alone but with vertical transmission, the presence of HDV results in either similar or lower levels of HBV prevalence as compared to HBV monoinfection.

Simulations showed that antiviral therapy reducing HDV infectivity will lead to a lower HDV prevalence than for therapies aimed at reducing HBV infectivity alone. In addition to reducing HDV prevalence, therapies reducing HBV infectivity will also reduce HBV prevalence. This means that HDV can be controlled using interventions directed towards HBV but not vice-versa. Therefore, HBV adult vaccination programs can play an important role in eradication of both viruses showing a primary effect on HBV prevalence and a secondary effect on HDV prevalence.

The current model has limitations as it excluded the transmission of HBV and HDV infection among injecting drug users (a high risk group). Additionally the model does not include change in infectivity according to age, and the sub-classification of dually infected individuals from superinfection and coinfection. Furthermore there were limited availability of HDV prevalence data that would affect the accuracy of predictions for this component of the model. It would be of interest to explore the economic costs of the different preventive measures along with intervention efficacy in a limited resource environment. Since most of the highly HBV and HDV endemic regions are in developing or under-developed countries, this approach will determine the best way to control HBV and HDV. Results from quantitative studies through mathematical models can then aid in policy decision making by governments.

In summary, we found that HDV can play an important role in the spread of HBV but has a negative effect on HBV prevalence. The sensitivity analysis of the extended model shows that vaccination coverage is the most influential factor in determining the fate of the HBV and HDV epidemics followed by the probability of perinatal transmission in the case of HBV prevalence. Thus HBV vertical transmission must not be ignored in any analysis. Although higher vaccination coverage of newborns should eliminate both HBV and HDV over a long time period, any short term strategy to eradicate HDV must include additional preventive measures such as HBV adult vaccination.
